# An Integrated Approach to Studying Rare Neuromuscular Diseases Using Animal and Human Cell-Based Models

**DOI:** 10.3389/fcell.2021.801819

**Published:** 2022-01-03

**Authors:** Timothy J. Hines, Cathleen Lutz, Stephen A. Murray, Robert W. Burgess

**Affiliations:** The Jackson Laboratory, Bar Harbor, ME, United States

**Keywords:** rare disease, charcot-marie-tooth disease, motor and sensory neuropathy, mouse model, iPSC model

## Abstract

As sequencing technology improves, the identification of new disease-associated genes and new alleles of known genes is rapidly increasing our understanding of the genetic underpinnings of rare diseases, including neuromuscular diseases. However, precisely because these disorders are rare and often heterogeneous, they are difficult to study in patient populations. In parallel, our ability to engineer the genomes of model organisms, such as mice or rats, has gotten increasingly efficient through techniques such as CRISPR/Cas9 genome editing, allowing the creation of precision human disease models. Such *in vivo* model systems provide an efficient means for exploring disease mechanisms and identifying therapeutic strategies. Furthermore, animal models provide a platform for preclinical studies to test the efficacy of those strategies. Determining whether the same mechanisms are involved in the human disease and confirming relevant parameters for treatment ideally involves a human experimental system. One system currently being used is induced pluripotent stem cells (iPSCs), which can then be differentiated into the relevant cell type(s) for *in vitro* confirmation of disease mechanisms and variables such as target engagement. Here we provide a demonstration of these approaches using the example of tRNA-synthetase-associated inherited peripheral neuropathies, rare forms of Charcot-Marie-Tooth disease (CMT). Mouse models have led to a better understanding of both the genetic and cellular mechanisms underlying the disease. To determine if the mechanisms are similar in human cells, we will use genetically engineered iPSC-based models. This will allow comparisons of different CMT-associated *GARS* alleles in the same genetic background, reducing the variability found between patient samples and simplifying the availability of cell-based models for a rare disease. The necessity of integrating mouse and human models, strategies for accomplishing this integration, and the challenges of doing it at scale are discussed using recently published work detailing the cellular mechanisms underlying *GARS*-associated CMT as a framework.

## Introduction

The number of disease-associated genes and pathogenic variants in those genes is rapidly increasing through the efforts of large-scale discovery programs such as the Centers for Mendelian Genomics (Posey et al., 2019). According to numbers from the World Health Organization, there are now as many as 8,000 rare diseases, many of which are genetic, and in aggregate, these disorders affect ∼1 in 15 people. However, there is no treatment for ∼90% of these disorders. With this increased rate of discovery and ongoing unmet clinical need comes the challenge of understanding how a given mutation leads to a given disease or phenotype. In some cases, this may be clear from the known function of the mutated gene, but more frequently, it requires a detailed exploration of the underlying genetic, cellular, and molecular mechanisms. Accomplishing such studies in patient populations is challenging for both ethical and practical reasons. It is even more challenging in the case of rare diseases, where access to patients is difficult and research materials such as surgical discards or postmortem samples are not available or are too rare to provide an experimentally feasible approach. In such cases, animal models can be powerful tools for *in vivo* experimentation. Animals can be used in sufficient numbers to provide well-powered studies and are more amenable to experiments that cannot be performed in humans. Furthermore, these animal models can be used to identify possible therapeutic strategies based on the identified disease mechanism, and to test these strategies in preclinical studies.

However, translation from animal models to patients is not always successful. Notable examples of this in neuroscience and neurology are failures to translate findings in animal models of amyotrophic lateral sclerosis (ALS) or Alzheimer’s Disease into successful clinical trials (Drummond and Wisniewski, 2017; Perrin, 2014; Philips and Rothstein, 2015). Sometimes this may reflect limitations of the model, such as when it does not fully recapitulate the human pathophysiology; for instance, a mouse model of CMT type 2D (CMT2D; *Gars* ^C201R^) develops a neuropathy with smaller axons, decreased nerve conduction velocity, grip strength, and body weight, but does not have frank axon loss. Due to the lack of axon degeneration, this would not be a good mouse model for testing therapies aimed at preventing axon loss, such as SARM1 inhibitors (Moss and Hoke, 2020). Fortunately, more severe CMT2D mouse models that do show axon degeneration exist (Table 1), and we will generate cell lines carrying multiple pathogenic *GARS* alleles. Alternatively, even if the mechanisms and targets are shared, the therapeutic tested in animals may not be equally effective in humans due to differences between mouse and human genetics, metabolism, or development. To address these issues and thus improve the translational potential of animal studies, it becomes necessary to also have a human experimental system. Induced pluripotent stem cells [iPSCs, (Takahashi et al., 2007)] potentially provide such a system, particularly as they can be derived directly from patients, or alternatively, specific patient mutations can be engineered into existing “healthy control” cell lines. In addition, iPSCs can be differentiated into many cell types of interest, such as motor neurons or cortical neurons, so that one is not restricted to studying neurodegeneration in skin fibroblasts or lymphocytes, for example. However, cell-based models also have inherent limitations, including the relative immaturity of their post-differentiation states, the comparatively isolated nature of the cell compared to their *in vivo* milieu, and the inevitable abstraction of the disease to a cellular phenotype (Santoso and Mccain, 2020). For example, patients do not see a neurologist because of reduced axonal transport, they go because of weakness or sensory deficits in their feet or hands, and whether improving axonal transport is truly tantamount to improving sensory/motor function *in vivo* is often a hopeful assumption.

**TABLE 1:** Current mouse models of *Gars*/CMT2D.

Mutation	Method of mutagenesis	Severity	Original publication
C201R	ENU-induced	Mild	Achilli et al., Dis Model Mech., 2009
ΔETAQ	CRISPR knock-in	Severe	Morelli et al., J Clin Invest., 2018
P278KY	Spontaneous	Severe	Seburn et al., Neuron, 2006
G240R	Adenovirus overexpression	Moderate	Seo et al., J Mol Histol., 2014
L129P	Adenovirus overexpression	Pain	Seo et al., J Korean Med Soc., 2014

Table 1 The C201R allele is not found in patients. While it causes marked weakness and reduced nerve conduction velocity, it has very little axon loss in motor or sensory nerves. The ΔETAQ allele is a mouse model recreating a *de novo* human mutation. It has a severe phenotype and pronounced axon loss in motor and sensory axons beginning at a few weeks of age. The P278KY allele is also not found in patients. It has a phenotype slightly more severe than ΔETAQ and can lead to premature mortality in an inbred genetic background. All three mutations are dominant and lead to a similar activation of the integrated stress response. The G240R and L129P mouse models were generated by viral overexpression of the mutant proteins. This has the advantage of efficiently testing pathogenicity for potential gain-of-function or dominant-negative alleles, but axonopathy was not characterized in these models.

We propose a solution to address the limitations of both animal models and cell-based models in which the findings in each are integrated to provide the best chance for successful translation of experimental approaches into clinical application. Here we will use the example of forms of Charcot-Marie-Tooth disease (CMT) to demonstrate how animal models have led to a better understanding of these diseases and have identified therapeutic targets and strategies that have been validated *in vivo*. We will also describe our strategy for using engineered human cell-based models to test whether the same pathogenic mechanisms and therapeutic strategies translate to a human system.

## tRNA Synthetase-Associated Forms of Charcot-Marie-Tooth Disease

The clinical hallmarks of CMT include degeneration and dysfunction in motor and sensory axons in the peripheral nervous system, leading to a length-dependent loss of sensation and muscle strength that is most pronounced in the feet and hands (Saporta and Shy, 2013). Mutations in close to 100 genes are now associated with CMT, suggesting a diverse array of mechanisms that can lead to a similar condition clinically (Timmerman et al., 2014; Laura et al., 2019). The largest gene family associated with CMT is the amino-acyl tRNA synthetase (aaRS) family, with dominant mutations in as many as six aaRS genes leading to forms of CMT (Wei et al., 2019). The first to be identified was Glycyl-tRNA synthetase (*GARS*), as the cause of CMT2D (Antonellis et al., 2003). Since that discovery in 2003, dominant mutations in Tyrosyl- (*YARS*), Alanyl- (*AARS*), Histidyl- (*HARS*), Tryptophanyl- (*WARS*), and possibly methionyl- (*MARS*) tRNA synthetases have been associated with forms of CMT (Jordanova et al., 2006; Latour et al., 2010; Gonzalez et al., 2013; Vester et al., 2013; Tsai et al., 2017). Most of these mutations lead to axonal forms of CMT, in which the motor and sensory axons themselves degenerate, without obvious involvement of the peripheral myelinating Schwann cells. However, mutations in *YARS* lead to an “intermediate” form of CMT (dominant intermediate CMT type C/diCMTC), with moderately reduced nerve conduction velocities, suggesting demyelination. Like many forms of CMT, the age of onset and severity vary both with gene and with the allele. For example, mutations in *GARS*/CMT2D patients may be incompletely penetrant, or lead to exclusively motor neuropathy, even in patients carrying the same allele (L129P) (Sivakumar et al., 2005). In contrast, at least one *GARS* patient was ascertained at 13 months of age with severe motor neuropathy resembling spinal muscular atrophy (Morelli et al., 2019). *GARS*/CMT2D patients are also distinctive in that they often have more severe symptoms in their hands than in their feet, whereas most forms of CMT are more severe in the lower extremities (Antonellis et al., 2003). Despite these idiosyncrasies, the neuropathies resulting from dominant tRNA synthetase mutations generally fit the clinical criteria for CMT.

How mutations in these genes lead to neuropathy was initially unclear. A primary question is whether the mutations cause a gain- or loss-of-function in the mutant protein? A second question is why the disease is specific to motor and sensory neurons, since the genes are expressed ubiquitously and involved in the “housekeeping” function of charging amino acids onto their cognate tRNAs as the first step in translation? We will summarize progress on the first point, as the genetic, cellular and molecular underpinnings of these disorders are becoming clearer, even if the second point of cellular specificity remains puzzling.

## Genetic Mechanisms Inform Gene Therapy Strategies

Many of the dominant mutations in tRNA synthetase genes that cause peripheral neuropathy also cause a reduction or loss of the mutant enzyme’s tRNA charging activity (Griffin et al., 2014). Furthermore, these enzymes form homodimers, and this is necessary for activity, leading to the possibility that there could be dominant negative effects in which the mutant subunit poisons the activity of the dimer. There are notable exceptions, such as *GARS* ^E71G^ and *YARS* ^E196K^, which retain most of their enzymatic activity in *in vitro* assays; however, loss of function *in vivo* could also result from mechanisms such as protein instability or mislocalization (Jordanova et al., 2006; Nangle et al., 2007).

However, data also argue against a simple loss of function and instead suggest a toxic gain-of-function (neomorphic) mechanism, in which the mutant gene product takes on a new, toxic function that cannot be corrected or out competed by the wild type gene product. First, in both humans and mice, a heterozygous null allele of *GARS* does not have a phenotype, indicating the neuropathy is not the result of a simple haploinsufficiency (Seburn et al., 2006; Oprescu et al., 2017). Furthermore, patients with recessive partial-loss-of-function mutations have severe, multisystem syndromic disorders in which peripheral neuropathy is not a prominent feature. If the dominant mutations led to peripheral neuropathy through a loss-of-function mechanism and thus served as a bellwether of dysfunction, then presumably these more severe recessive syndromes would also include peripheral neuropathy as an early and severe outcome. However, perhaps the most convincing data supporting a neomorphic activity comes from animal models. In *Drosophila*, the transgenic overexpression of mutant *GARS* or *YARS* leads to axon degeneration and CMT-relevant phenotypes, whereas overexpression of the wild-type genes has no effect, and the endogenous fly *Gars* and *Yars* are still functional (Storkebaum et al., 2009; Grice et al., 2015; Niehues et al., 2015). Indeed, levels of tRNA charging activity in tissue homogenates were never reduced below wild-type levels, arguing against a dominant negative effect leading to loss of function (Niehues et al., 2015). In a reciprocal experiment in mice, transgenic overexpression of wild-type *GARS* did not rescue the neuropathy phenotype of dominant *Gars* mutations (Motley et al., 2011). The failure of robust overexpression of the wild-type protein to rescue is most consistent with a neomorphic activity, whereas a loss of function or even dominant negative should be at least partially corrected by excess wild-type expression.

Confirming the genetic mechanism is critical for designing gene therapies, such as gene replacement or gene knockdown. As an example in neuromuscular diseases, spinal muscular atrophy can be treated by delivery of the wild-type *SMN1* gene by AAV9-mediated delivery to motor neurons (Mendell et al., 2017). This is now an approved therapeutic approach for SMA. A similar gene replacement strategy was shown to be effective in a mouse model of CMT type 4J, caused by recessive mutations in *Fig4* (Presa et al., 2021). However, the *Gars* transgenic mouse result showing that transgenic overexpression of the wild-type gene does not correct the phenotype argues against a gene replacement approach for *GARS*/CMT2D. Instead, the fact that heterozygous null mice and human carriers are healthy suggests that allele-specific knockdown of the mutant gene product while preserving the expression of the wild type allele should be an effective strategy. If done with complete efficiency and specificity, this strategy would effectively reproduce the heterozygous null situation and eliminate any neomorphic effect of the mutant protein.

This was indeed shown to be an effective strategy in mouse models (Morelli et al., 2019). Two alleles of *Gars*, including one carrying an engineered human disease-associated allele, were treated with allele-specific RNAis that precisely matched the mutant allele but mismatched with the wild-type mRNA. The RNAi was generated from an engineered mir30 microRNA shuttle that was expressed behind an RNA PolIII U6 promoter. This was delivered to the nervous system *in vivo* using self-complementary AAV9. When delivered at birth, 2–3 weeks before the onset of a neuropathy phenotype, the disease was almost completely prevented, and consistent with the perdurance of expression from AAVs, beneficial effects lasted at least 1 year. Benefit was still obtained with delivery after the onset of neuropathy, but decreased the later treatment was started. Whether the declining efficacy is because the phenotype is irreversible or whether it is because AAV9 spread and transduction efficiency decreases with age is unclear.

The preclinical gene therapy studies described above provide the *in vivo* proof-of-concept confirmation that eliminating the mutant gene product produces clinical benefit. It is likely that similar effects could be produced with other approaches, such as allele-specific antisense oligonucleotides. However, target sequence-specific strategies require extensive research and development and regulatory efforts for each new sequence entity. An alternative approach that may provide a “generic” strategy for any *GARS* mutation (or any dominant neomorphic mutation) is to knockdown all transcripts, mutant and wild type, and to replace them with a knockdown-resistant wild-type cDNA, ideally delivered in the same vector as the knockdown RNAi so that any cell getting the knockdown also gets the replacement. Such a strategy has been successfully executed in mice for alpha-1 antitrypsin (Li et al., 2011), and is being explored for *GARS*/CMT2D.

## Cellular and Biochemical Mechanisms Suggest Therapeutic Strategies

Axonal peripheral neuropathies are generally considered to be problems of axon degeneration and clearly axon degeneration disconnects neurons from their targets, whether these are sensory endings in the periphery, or muscles in the case of motor neurons. However, in *Gars*/CMT2D mouse models, it was also shown that while axons maintained neuromuscular junctions (NMJ), but had perturbed synaptic morphology and function at the NMJ (Spaulding et al., 2016). In fact, this was true even in proximal muscles that had little frank denervation. Therefore, part of the neuromuscular phenotype of these mice could be considered a myasthenia and not just an axonal neuropathy. This raises the possibility that therapeutics designed to enhance synaptic transmission at the NMJ may be beneficial in CMT2D. Whether defects in NMJ transmission are commonplace in other axonal neuropathies remains to be tested, but it stands to reason that the axons that remain intact in these diseases may not be functioning perfectly, and that this would manifest itself at the NMJ. This possibility is now being explored in a clinical trial in CMT patients (NCT04980807). Improving synaptic transmission at the NMJ is unlikely to address the core pathophysiology of these disorders and would therefore not necessarily be expected to slow progression or promote regeneration. However, it may improve function for patients who already have the disease, and a further exploration of NMJ involvement in different forms of CMT is warranted in both mouse models and patients.

Investigations of the neomorphic activities of mutant tRNA synthetases have focused on novel interactions mediated by the mutant protein that are not found in the wild-type protein. Mutations in both *GARS* and *YARS* (also known as GlyRS and TyrRS for proteins) result in conformational changes that potentially expose new protein surfaces, enabling novel interactions (Nangle et al., 2007; Xie et al., 2007; Blocquel et al., 2017). One intriguing interaction identified is the binding of Neuropilin-1 (NRP1) to mutant GlyRS (He et al., 2015). NRP1 is a developmental receptor that has both semaphorins and vascular endothelial growth factors (VEGFs) as ligands. Both semaphorin and VEGF signaling have neurodevelopmental roles, but the binding of mutant GlyRS specifically competes with VEGF. VEGF overexpression mitigates some aspects of the neuropathy seen in Gars mutant mice. The necessity of GlyRS binding NRP1 for the disease mechanism leading to neuropathy is called into question by the failure of NRP1 to bind ΔETAQ, a four amino acid internal deletion in GlyRS that causes a severe, early onset neuropathy in both mice and humans (Morelli et al., 2019). Nonetheless, several other neuropathy-associated mutant GlyRS proteins bind NRP1, as do CMT2N-associated alleles of *AARS*, suggesting that it may in some way be contributing to the disease severity and pathogenesis (He et al., 2015; Sun et al., 2021). This also suggests that small molecules to block this interaction or antibodies to clear mutant GlyRS proteins could be therapeutic strategies.

**FIGURE 1:**
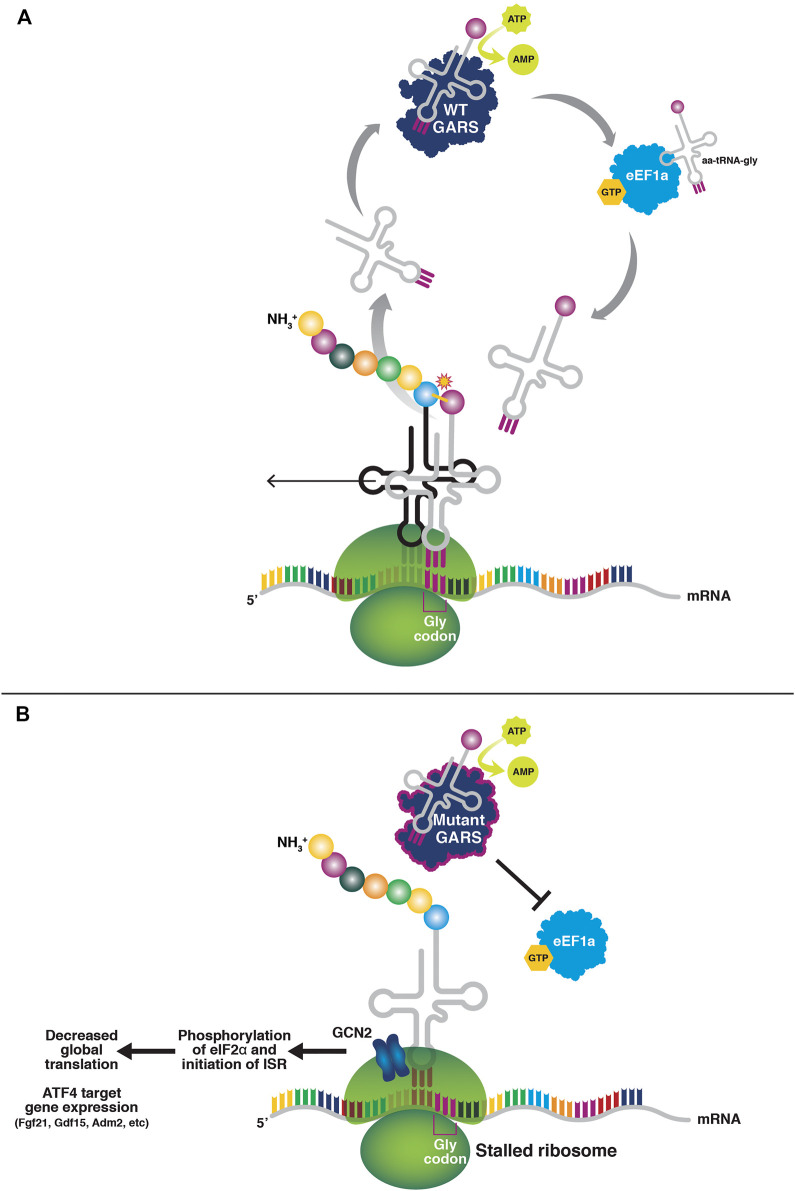
tRNA sequestration by mutant tRNA synthetases. **(A)** In normal tRNA charging, the amino acid binds the tRNA synthetase and is coupled with ATP to form an aminoadenylate intermediate. The amino acid is then charged onto the 3′ end of the cognate tRNA. The amino acid-charged tRNA is shuttled to the ribosome by eEF1A to participate in translation. **(B)** Mutant tRNA synthetases do not release the tRNAs to eEF1A, thus resulting in a paucity of charged tRNAs for translation elongation and subsequently ribosome stalling at Glycine codons (in the case of mutant glycyl tRNA-synthetase). The stalled ribosomes activate GCN2 and the integrated stress response, resulting in a suppression of global cap-dependent translation through eIF2α phosphorylation, and activation of ATF4 target gene expression.

Recent results suggest an alternative biochemical mechanism that may extend more logically across the tRNA synthetase-associated neuropathies (Figure 1). The mutant forms of GlyRS and TyrRS bind their cognate tRNAs, but have a much slower off-rate, effectively resulting in the sequestration of the tRNAs and precluding their transfer to the ribosome to participate in translation. Consistent with tRNA sequestration being considered a toxic, gain-of-function activity, overexpression of mutant forms of *GARS* in HEK293 cells resulted in ribosome stalling at Glycine codons, as expected if the mutant enzymes were expressed at high enough levels to sequester tRNAs in cell types where the tRNAs are not typically limiting (Mendonsa et al., 2021). In addition to these biochemical findings, this mechanism is also supported genetically for *GARS*. The overexpression of tRNA^Gly^ rescues neuropathy and axon degeneration phenotypes in both mouse and fly models of *GARS*/CMT2D (Zuko et al., 2021). This is in contrast to overexpression of the wild-type synthetase in mice (Motley et al., 2011), which despite being active does not rescue, and would have a paucity of tRNA substrate to charge under this mechanistic model.

The result of tRNA sequestration is stalling of elongating ribosomes at Glycine codons. Stalled ribosomes are potent activators of the kinase GCN2 and subsequently, the integrated stress response (ISR) (Inglis et al., 2019). Interestingly, mitochondrial dysfunction is also an ISR activator, and GlyRS is a bifunctional enzyme that also encodes the mitochondrial glycyl-tRNA synthetase (Turner et al., 2000). In fact, patients with recessive loss-of-function *GARS* mutations experience a multisystem developmental syndrome with mitochondrial abnormalities (Mcmillan et al., 2014; Oprescu et al., 2017). While mitochondrial dysfunction has not been explicitly ruled out as an activator of the ISR in mouse models of CMT2D, the fact that TyrRS (and all of the other known CMT-associated synthetases, AlaRS, HisRS, MetRS, and TrpRS) is cytosolic and not mitochondrial, yet CMT-associated *YARS* mutations in mice and humans cause a similar phenotype, argues against mitochondrial involvement as the primary disease mechanism.

The ISR is beneficial under many forms of cellular stress (Pakos-Zebrucka et al., 2016). For example, amino acid starvation also causes uncharged tRNA and stalled ribosomes, both of which activate GCN2. The result is the phosphorylation of elongation initiation factor 2-alpha (eIF2α). This globally suppresses cap-dependent translation. In addition, the translation of the stress-response transcription factor, ATF4, is increased through an upstream open reading frame mechanism that regulates its synthesis (Vattem and Wek, 2004). ATF4 promotes the expression of cell-type-specific stress response genes. Thus, by suppressing cap-dependent translation and enhancing expression of stress response genes, the ISR can be beneficial in the face of a transient stress such as amino acid starvation.

Importantly, in tRNA synthetase-associated CMTs, the ISR is not being activated by a transient stress, but instead by the chronic sequestration of tRNAs. In an analogous case of neurodegeneration caused by ribosome stalling during translation elongation due to a combination of a tRNA^Ala^ mutation and loss of GTPBP2, which rescues stalled ribosomes from mRNAs, the inhibition of GCN2 and the ISR is detrimental, exacerbating the associated neurodegeneration (Ishimura et al., 2014; Ishimura et al., 2016). However, in the case of *Gars*/CMT2D mouse models, genetically deleting or pharmacologically inhibiting GCN2 not only eliminates ISR activation, but also greatly mitigates the severity of the neuropathy phenotype (Spaulding et al., 2021). That an experimental drug was also effective in mitigating neuropathy in the Gars mice speaks to the translational potential of inhibiting GCN2 to treat dominant tRNA synthetase-associated peripheral neuropathies.

Although these studies provide a biochemical mechanism through which tRNA synthetase mutations may act and identify GCN2 as a therapeutic target, they do not directly resolve whether the neuropathy results from the further decrease in protein synthesis that results from phosphorylation of eIF2α, from a toxic effect of ATF4 target-gene expression, or from a combination of these ISR actions. Nor does this biochemical mechanism explain the cell-type specificity of the disease and why only alpha motor neurons and a subset of sensory neurons are affected. The finding that the ISR is similarly activated in both *Gars* and *Yars* mutant mice suggests that this mechanism may be in play across the tRNA synthetase-associated neuropathies, though this remains to be tested for mutations in *HARS*, *WARS* and *AARS*. Furthermore, though the *Gars*/CMT2D mouse models accurately recapitulate the human disease, it also remains to be determined whether the same ISR mechanism is active in human motor and sensory neurons. Circulating levels of one prominently upregulated, secreted ATF4 target gene, GDF15, were elevated in patients with tRNA synthetase mutations; however, GDF15 was also elevated in *PMP22*/CMT1A patients (Spaulding et al., 2021). This is interesting and may suggest GDF15 is a general biomarker for multiple forms of CMT, but it makes the direct relevance to ISR activation in the tRNA synthetase mutations less definitive. Confirming activation of the ISR and further exploring the biochemical mechanisms and cellular specificity of the disease are all questions that can be efficiently addressed using human cell-based models.

## Human Cell-Based Models of tRNA Synthetase-Associated Neuropathy

Rare diseases present a challenge for iPSC derivation and experimentation. Namely, patients are challenging to find and generating an experimentally robust battery of patient-derived cell lines accounting for multiple genes and multiple alleles of each gene, as well as other biological variables such as sex, age, and ethnicity may simply not be feasible. Other technical variables, such as the cell type from which the iPSCs were derived, the protocol for reprogramming those cells, and the culture conditions may all create additional complications, particularly if cell lines are being sourced globally and are not being generated through a centralized facility or research program. While there are large research programs attempting to overcome these challenges by increasing the number of control and mutant cell lines being compared, such as AnswerALS (https://www.answerals.org/), for most laboratories, it would quickly lead to even the simplest experiments becoming unwieldy.

**FIGURE 2:**
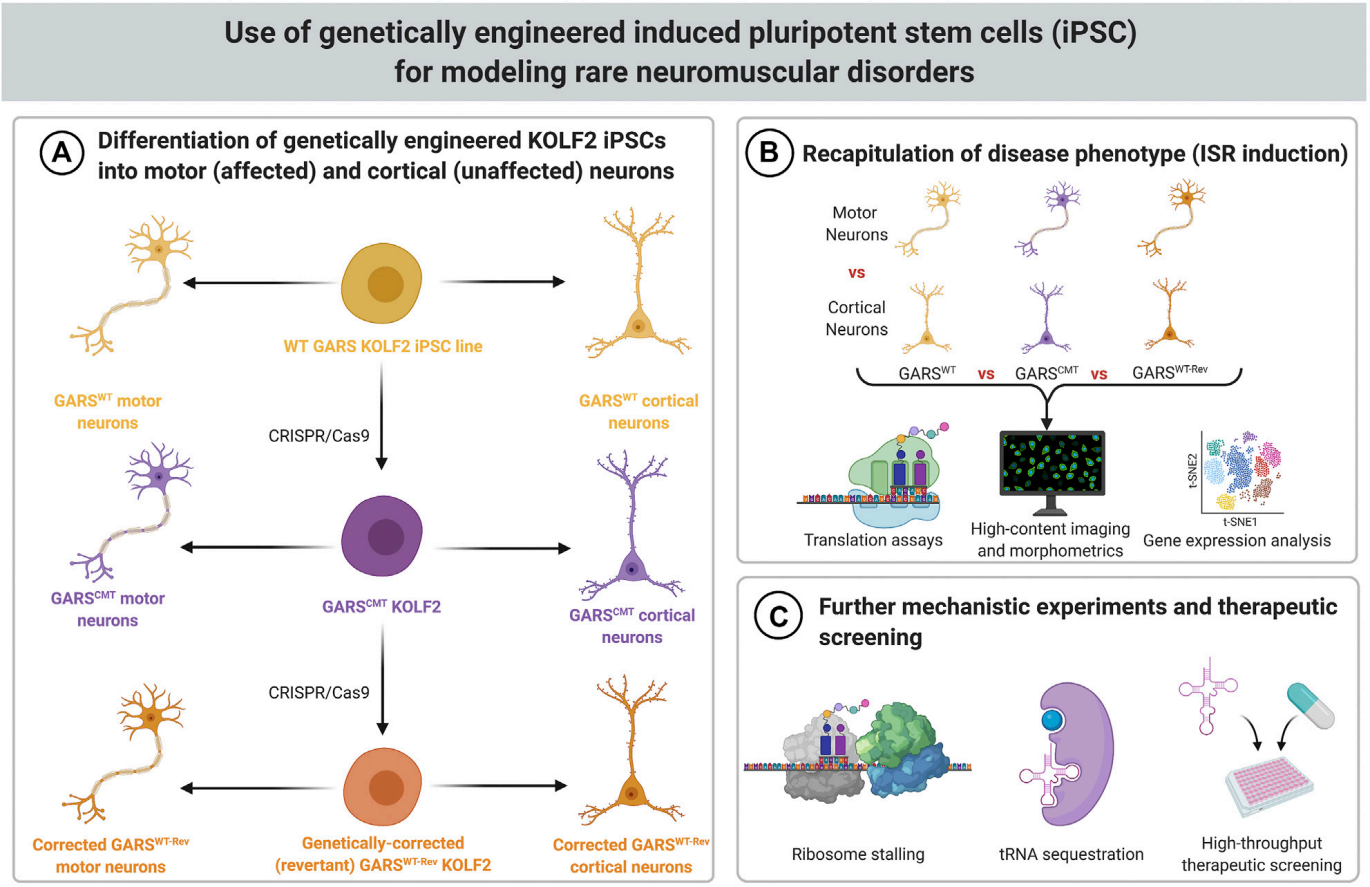
Schematic for modeling rare neuromuscular disorders using genetically engineered iPSCs. **(A)** KOLF2 iPSCs genetically engineered to carry CMT-associated *GARS* mutations can be differentiated into motor neurons, which are affected by CMT, and cortical neurons, which are not affected by CMT. The wild-type parental cells and revertant cells (which have had the introduced *GARS* mutation corrected back to WT) can be used as controls. **(B)** Translation assays, high content imaging and morphometrics, and gene expression analysis can be used to determine if the ISR is activated in *GARS* mutant motor neurons. **(C)** If motor neurons have the expected phenotype (ISR induction), then they can be used for further mechanistic experiments, such as ribosome stalling assays and analysis of biochemical properties of tRNA sequestration. These cells can also be used for high-throughput screens of therapeutics.

One possible solution to these practical and technical challenges is to use engineered cell-based models. Efficient genome editing in human cell lines makes the introduction of most human disease-associated mutations in a “healthy control” iPSC line relatively straightforward (Figure 2). For rigor, these mutations can be introduced into multiple cell lines to begin to capture human genetic diversity. Cell-based models can also be engineered so that they can be reverted, such that both the parental cell line and the revertant can serve as controls for the mutated cell line. The use of revertant cells is most important when the pathogenicity of the allele is uncertain, or if only a single pathogenic variant is known or available, as is sometimes the case for rare diseases. For patient-derived cells, revertant cells would control for genetic background by fixing the causative mutation. In the engineered cells, revertant lines serve as a stringent control for the targeting and gene editing process required to introduce the mutation. The use of revertant strains may be unnecessary if a large number of independent disease-associated and healthy control cell lines are available. In such a case, rigor is provided by consistency across independent cell lines and the use of revertant cell lines could again make the approach unwieldy.

Another advantage of the engineered cell lines is that multiple, related mutations can be introduced independently into the same cell line. Thus, cell-based models, each carrying a disease-associated allele of *GARS*, for example, can be generated and compared to otherwise isogenic lines carrying mutations in other tRNA synthetase genes such as *YARS*, *AARS*, *WARS* or *HARS*. Much like engineering disease models in a single inbred mouse strain allows for consistency and an “apples-to-apples” comparison of phenotypes and mechanisms, these cell-based models should vary only in their disease-associated mutation or allele that they carry. This approach of introducing disease-associated variants into a well-defined cell line is being used to study genes and variants associated with Alzheimer’s and related dementias, through the iPSC Neurodegenerative Disease Initiative (iNDI) program, for example (Ramos et al., 2021).

In addition to the obvious advantage of demonstrating the same pathophysiological mechanisms are engaged in a human disease model, iPSC-based models also have advantages for exploring disease mechanisms (Figure 2). In the case of *GARS*/CMT2D, the human disease is a predominantly motor neuropathy, and there are no reproducible pleiotropic effects. In mouse models, the activation of the integrated stress response is specific to alpha motor neurons and a subset of sensory neurons, and presumably this indicates that ribosome stalling is restricted to these cell types. Consistent with this, when the ribosome rescue factor *Gtpbp2* was genetically deleted, the neuropathy phenotype of *Gars* mice become more severe, providing indirect genetic evidence for ribosome stalling; however, the ISR did not appear in other cell types even after the ribosome rescue factor was deleted (Zuko et al., 2021).

This specificity of ribosome stalling presents a challenge for detailed studies of this mechanism *in vivo*. Motor neurons represent a small fraction of the total cells in the spinal cord, and ribosomes isolated from bulk tissue complicate interpretation of assays such as ribosome footprinting, in which RNase digestion removes mRNA except those regions protected by a stalled ribosome. Such assays could identify particular mRNAs or particular Glycine codons that are particularly sensitive to ribosome stalling in motor neurons. The latter may identify specific anticodon tRNAs that are most limiting after sequestration by the mutant synthetase. Since iPSCs can be differentiated to motor neurons with high efficiency, resulting in a relatively homogeneous population of cells, experiments such as ribosome footprinting will be much more straightforward in cell-based models, where ribosomes can be rapidly isolated from the bulk culture. It is even possible to generate specific sub-populations of motor neurons, such as cranial motor neurons, or limb-innervating lateral motor column motor neurons (Amoroso et al., 2013; An et al., 2019). This is additional degree of differentiation is potentially useful given that CMT2D primarily affects spinal alpha motor neurons.

The homogeneity and scalability of culture systems will also be an advantage for probing the cell-type specificity of ISR activation. The same iPSCs can be differentiated into motor neurons, where we expect to see ISR activation, or into a closely related cell type, such as cortical neurons, or even an unrelated cell type such as cardiac myocytes, where we do not expect to see ISR activation. Thus, detailed characterization of gene expression, codon usage, tRNA expression, and other parameters can be performed to see if they may explain why motor neurons exhibit ribosome stalling and ISR activation and other cell types do not.

## Enhancing Cell Maturity and Exploring Cell Biological Mechanisms

While the homogeneity of an iPSC-based system can be a great advantage for the types of studies detailed above, it may also be beneficial for us to use a co-culture system with motor neurons and other relevant cell types. For example, human iPSC-derived co-cultures of astrocytes and motor neurons were recently used in a study showing that astrocytes exhibit non-cell autonomous effects on motor neurons in an *in vitro* ALS model (Zhao et al., 2020). For studies on neuromuscular disease, co-cultures of iPSC-derived motor neurons with skeletal muscle cells (such as those derived from C2C12 mouse myoblasts, primary human-derived myoblasts, or differentiated from hiPSCs, with varying degrees of difficulty and availability) to simulate a simplified version of the neuromuscular system present *in vivo*, and allowing for assays of NMJ function (Demestre et al., 2015; Picchiarelli et al., 2019; Lin et al., 2020; Yoshioka et al., 2020; Stoklund Dittlau et al., 2021). Furthermore, organoid culture systems allow for the differentiation of multiple cell types to form a miniature version and experimentally approachable model of the tissue of interest (Vieira de Sa et al., 2021). Recently, iPSC-derived human sensorimotor organoids, which contain motor and sensory neurons, skeletal muscle, astrocytes, microglia, and vasculature, have been used to assess how familial ALS mutations affect NMJ and muscle function *in vitro* (Pereira et al., 2021).

In addition to single-well co-cultures, a variety of multiple compartmentalized culture platforms are also available. These house the motor neurons and muscle cells in separate chambers with microgrooves between them, which allows the motor axons to extend through the grooves to form a spatially and chemically isolated synapse on the muscle cells (Santhanam et al., 2018; Altman et al., 2019; Ionescu and Perlson, 2019). An added benefit of this system is that treatments can be restricted to either the cell body compartment or the axon terminal/muscle compartment if using microfluidic chambers. A recently published protocol describes the creation of an “NMJ chip”—a 3D culture system with neurons and myocytes in separate compartments which form functional NMJs and allows imaging and functional assessment of the motor unit. The myocyte compartment contains two pillars made of flexible polydimethylsiloxane (PDMS) to which the myocytes attach to form a skeletal muscle bundle. Muscle contractile force is measured based on the flexion of the PDMS pillars (Osaki et al., 2018; Osaki et al., 2020). One advantage of the single-well co-culture systems is that they do not require specialized culture dishes; however, the compartmentalized system allows for localized treatment of axon terminals or neuronal cell bodies.

An intriguing hypothesis regarding the motor and sensory neuron sensitivity to tRNA synthetase mutations and ribosome stalling relates to translation in distal axons. In both mice and humans, it is the longest and largest axons that are most affected (Antonellis et al., 2003; Sleigh et al., 2014). Unfortunately, this invokes many possible mechanisms. Presumably these cells are more metabolically active to maintain their resting potential with the large cell volume and are more dependent on efficient axonal transport to maintain their distal processes, to name just two logical possibilities. However, they may also be more dependent upon local protein synthesis in the distal axon to maintain processes such as mitochondrial function that may not be sufficiently supported by transport of components from the cell body. The evidence supporting the importance of local protein synthesis in axons is increasingly convincing (Baleriola and Hengst, 2015; Spaulding and Burgess, 2017; Fernandopulle et al., 2021). Axonal translation could intersect with the tRNA sequestration mechanism described above for tRNA-synthetase-associated neuropathies if the tRNAs become limiting, and therefore most susceptible to sequestration, in the distal axon. This is very challenging to test *in vivo*, but is straightforward to test *in vitro* using compartmentalized culturing systems, which would allow levels of both mRNAs and tRNAs in axons to be compared to levels in cell bodies. In addition, manipulations such as GCN2 inhibitors could be applied specifically to axons or cell bodies to see if one compartment or the other was the primary site of ISR activation. It is unclear how such experiments could be done *in vivo*.

Thus, while caveats remain such as the maturity of the cells in culture and whether the ISR will in fact be activated *in vitro*, the iPSC cell-based models have the potential to greatly expand the level of mechanistic studies regarding translation and ribosome stalling. The cells also offer a rapid system for testing GCN2 and ISR inhibitors, with cellular level readouts of ISR such as levels of phospho-eIF2α, phospho-GCN2, or ATF4, even if frank axon degeneration is not evident in the culture system. Importantly though, even though the iPSC model may have also led to the discovery of ISR activation, the *in vivo* animal models were necessary to show that GCN2/ISR inhibition was an effective treatment for neuropathy given that in other neurodegenerative conditions it has been found that activation of the ISR is actually neuroprotective (Ishimura et al., 2016; Sidoli et al., 2016). With this knowledge, the studies in the cell-based models can be interpreted with the sound assumption that inhibiting the ISR *in vitro* should be beneficial for treating neuropathy *in vivo*.

## Scalability

The findings and proposed research above present a template for studies of rare neuromuscular diseases using a combination of animal and human cell-based models. Animal models reveal genetic mechanisms, leading to gene therapy strategies that can then be tested for *in vivo* efficacy in those same models. Similarly, animal models can reveal mechanistic insights that may again suggest therapeutic strategies, such as the identification of ISR activation as a contributor to the pathophysiology and GCN2 as a drug target for *GARS*/CMT2D. Human cell-based models can be used to confirm those same mechanisms. They also provide a higher throughput platform of compound testing and a more homogeneous system for biochemistry and -omics studies, at least compared to a cell type such as motor neurons, where the cells of interest represent a small proportion of cells resident in the tissue. However, this example is still incomplete, as the cell-based models are in their early stages of analysis, and it already represents 18 years of work if the identification of *GARS* as the causative gene for CMT2D is considered the start date. In light of this, what are the prospects for streamlining such studies and doing them at scale?

With current technologies and infrastructure, it is feasible to generate hundreds of genetically modified mouse strains every year. This is exemplified by large programs such as the Knockout Mouse Phenotyping Program (KOMP2), and even small-scale programs such as the Resource for Research on Peripheral Neuropathy (RRPN, NINDS R24 NS098523), which was able to generate multiple mouse models of human disease in any given year at the scale of a single lab and modest funding. The KOMP2 phenotyping pipeline was established to capture data for gene knockouts for the entire mammalian genome, and is (intentionally) broad and not deep for any specific physiological system (Brown et al., 2018). A given phenotype domain, such as neuromuscular performance, may be covered by only one or two tests, and is not supported by detailed physiology, histology, or biomarker analysis. Therefore, high-throughput pipelines such as KOMP2 are best suited for establishing baseline data to describe gene function, and as discovery tools to identify and prioritize disease-relevant mutant phenotypes for more extensive analysis. The output from the program has been significant. For example, data from the International Mouse Phenotyping Consortium (IMPC), to which KOMP2 contributes, has revealed that ∼40% of all lines generated have phenotypic overlap with human disease and that for a significant majority, the IMPC model was the first mutant mouse line reported for the gene-disease (Meehan et al., 2017). IMPC data has been mined to identify novel disease-relevant phenotype associations for deafness, metabolic disease, and skeletal abnormalities, among others (Bowl et al., 2017; Rozman et al., 2018; Swan et al., 2020). Although the KOMP2 program has focused on generating null alleles, repurposing this capacity for more precise and/or disease-relevant alleles is quite feasible, and would take full advantage of the engineering and production platforms investments made to support these programs.

Despite the potential benefits of high-throughput phenotyping for target discovery and disease gene prioritization, a significant phenotyping gap remains to not only make them convincing human disease models, but also to provide the necessary information for future mechanistic and preclinical studies. Mechanistically, parameters such as the cell-autonomy and tissue-specificity of the pathophysiology, or whether the disease is developmental or degenerative need to be determined, to name just two important considerations. For preclinical studies, the age of onset, potential for sex-specific differences, the most informative outcome measures, and the rate of progression all need to be determined before any therapeutic testing can begin. While these analyses can be pipelined to some extent by running a standard battery of tests, each mouse model, like each human disease, inevitably has specific characteristics that need to be addressed, requiring a deviation from the script. All of this requires a certain amount of bespoke analysis and represents a somewhat slow and somewhat trial-and-error process that reduces throughput.

Generating *in vivo* animal models through other technologies such as viral delivery of mutant genes is also an option to increase throughput. This requires the generation of many different allele sequences and packaging these into viruses for *in vivo* delivery, but these technologies are well established. Constraints on this system are the size of constructs that can be packaged into viral vectors, and that such overexpression systems, at least in a wild-type background, will only model gain-of-function or dominant-negative alleles. Nonetheless, this approach has been used effectively in *C9ORF72* ALS, and has been attempted for *GARS*/CMT2D (Table 1), although axonopathy was not described (Lee et al., 2014; Seo et al., 2014; Chew et al., 2015; Herranz-Martin et al., 2017). While such viral approaches allow a large number of alleles to be generated, phenotyping is still required.

Cell-based models may offer greater opportunity to increase scale. For diseases with relatively large patient populations, generating a large and diverse patient-derived iPSC collection may be feasible. This strategy is currently being used for sporadic forms of ALS (SALS) by the Answer ALS Research Project, who hope to gather and characterize iPSCs from 1000 SALS patients to find biomarkers of the disease, determine why motor neurons are the primary affected cell type, and what role genetic background plays in these sporadic cases (https://www.answerals.org/). For rarer mutations, engineering disease-associated mutations into control iPSC lines is feasible at reasonable scale. The iNDI program is an example, where over 100 alleles of Alzheimer’s and related dementia genes will be engineered into a “healthy control” iPSC line and then phenotyped in an automated platform (Ramos et al., 2021). The approach of engineering mutations into an otherwise isogenic background has other potential benefits, as described above. Provided the cell types of interest have established differentiation protocols, experiments can largely be done using high-content imaging platforms and automated morphometry and related assays. Similarly-omics studies are highly feasible in such models. Additional assays, such as mitochondrial function, organelle trafficking, etc. can also be done with reasonable throughput. Cell lines do not necessarily have to recapitulate the disease to be useful. For example, if iPSCs engineered to carry *GARS*/CMT2D mutations show ISR activation when differentiated to motor neurons, this will be a useful model even if they do not show axon degeneration. However, this requires sufficient understanding of the disease mechanism to accurately interpret these secondary phenotypes. Ultimately, gaining that level of understanding may require an *in vivo* model, which may again limit the scale of these studies. Nonetheless, the cell-based models may identify mechanisms to be tested *in vivo*, improving efficiency, and once such mechanisms are identified, the cell-based systems provide an efficient platform for testing interventions. Approaches such as transcriptomics or biomarker analysis may also provide ways to readily bridge cell-based models with *in vivo* results and predictions.

An example of the successful use of an iPSC-based platform to interrogate disease mechanism with the goal of bringing a therapeutic to the clinic was done using cell-based models of amyotrophic lateral sclerosis (ALS). Patient iPSC-derived motor neurons with a disease-causing mutation in superoxide dismutase 1 (*SOD1*) were found to exhibit hyperexcitability. This was similar to findings from clinical neurophysiological studies (Wainger et al., 2014). This phenotype was abolished when the *SOD1* mutation was reversed in the cells using CRISPR genome editing (Kiskinis et al., 2014). In addition, it was found that the hyperexcitability was conserved across multiple forms of ALS using iPSC-derived motor neurons from patients with mutations in *C9ORF72*, *FUS*, and different *SOD1* alleles (Wainger et al., 2014). The hyperexcitability was successfully corrected and motor neuron survival increased when treated with the drug ezogabine (also known as retigabine), a Kv7.2/3 potassium channel agonist used to treat epilepsy (Wainger et al., 2014). Data from these studies led to a recently published double-blind, placebo-controlled phase 2 clinical trial using ezogabine to reduce motor neuron hyperexcitability in both familial and sporadic ALS patients. Notably, this drug was not tested in the canonical *SOD1* transgenic mouse model of ALS before moving to clinical trials, as the human cell-based data was deemed sufficient (Mcneish et al., 2015; Wainger et al., 2021).

## Addressing Scalability

While the challenges of creating, validating and using disease models at scale are significant, they are at least somewhat addressable. CRISPR/Cas9 technology allows for the rapid creation of patient-based mutations in mice. Mutations in any one disease gene can be strategically prioritized based on criteria, such as common mutations based on founder affects or mutations that may be more amenable to genomic based therapeutic strategies, such as ASOs, read through drugs, or gene therapy. By creating an allelic series for a particular disease-causing gene, potential therapeutics can be properly aligned with specific mutations. Phenotyping of these mouse models can also be strategically prioritized by matching prominent clinical features of the patients with a battery of tests in mice that would zero in on these deficiencies. The creation of these mini-pipelines allows for a first pass, direct interrogation of translationally relevant preclinical phenotypes that can be rapidly evaluated with specific therapeutics. Keeping in mind that ultimately, not every clinical feature in a patient may be addressable. The goal of many therapeutics is not curative but is to alleviate some of the features with the goal of improving the quality of life for patients.

Approaches for achieving this are being piloted by programs such as the JAX Center for Precision Genetics. This NIH-funded program seeks to better integrate current efforts in human genetics, disease modeling both in mice and in other systems, and preclinical studies to lessen the time from gene discovery to clinical trial. An important part of this effort is bioinformatics, to evaluate the feasibility/necessity for making a model and for optimizing the probability that such a model will be successful. For example, if a disease mechanism or relevant biological pathway is known, are some strains of inbred mice more or less likely to be good genetic backgrounds for producing valid disease models based on known differences in those pathways across mouse strains? As described above, the genome engineering capacity, although ultimately finite, is unlikely to be limiting. Instead, the phenotyping capacity for validation of disease models, as well as evaluating preclinical studies are still the most labor intensive and time-consuming phase. It is not making the model that is the challenge in 2021, it is proving it is a valid model and using it productively that takes time and effort.

## Summary

We have provided an example of a rare neuromuscular disease, inherited peripheral neuropathy caused by dominant tRNA synthetase mutations, as a demonstration of how mouse and human cell-based models can be integrated to understand disease mechanisms, test therapeutic approaches, and improve the likelihood of successful translation to clinical practice. Both *in vivo* models such as mice and human cell-based models have advantages and limitations. Relying on either alone has risks, so the clear solution is to incorporate both approaches and thus hopefully minimize those risks. For each, the challenge currently is not genetically engineering the model, it is the effort of validating the model as disease-relevant and using it in well-designed preclinical studies. This is being successfully done for many diseases besides inherited peripheral neuropathies, but it remains a challenge to do it at a scale that will rapidly deliver results for the ∼7,000 rare diseases now identified.
